# Structure of Dihydrochalcones and Related Derivatives and Their Scavenging and Antioxidant Activity against Oxygen and Nitrogen Radical Species

**DOI:** 10.3390/molecules16021749

**Published:** 2011-02-21

**Authors:** Alexandre L. A. Bentes, Rosivaldo S. Borges, Waldinei R. Monteiro, Luiz G. M. de Macedo, Cláudio N. Alves

**Affiliations:** Laboratório de Planejamento e Desenvolvimento de Fármacos, Universidade Federal do Pará, CP 11101, 66075-110, Belém, PA, Brazil

**Keywords:** DFT, antioxidant activity, SAR, dihydrochalcone derivatives, ionization potential, bond dissociation enthalpy, ROS and RNS

## Abstract

Quantum mechanical calculations at B3LYP/6-31G** level of theory were employed to obtain energy (E), ionization potential (IP), bond dissociation enthalpy (O-H BDE) and stabilization energies (ΔE_iso_) in order to infer the scavenging activity of dihydrochalcones (DHC) and structurally related compounds. Spin density calculations were also performed for the proposed antioxidant activity mechanism of 2,4,6-trihydroxyacetophenone (2,4,6-THA). The unpaired electron formed by the hydrogen abstraction from the phenolic hydroxyl group of 2,4,6-THA is localized on the phenolic oxygen at 2, 6, and 4 positions, the C_3_ and C_6_ carbon atoms at *ortho* positions, and the C_5_ carbon atom at *para* position. The lowest phenolic oxygen contribution corresponded to the highest scavenging activity value. It was found that antioxidant activity depends on the presence of a hydroxyl at the C2 and C4 positions and that there is a correlation between IP and O-H BDE and peroxynitrite scavenging activity and lipid peroxidation. These results identified the pharmacophore group for DHC.

## 1. Introduction

**Figure 1:**
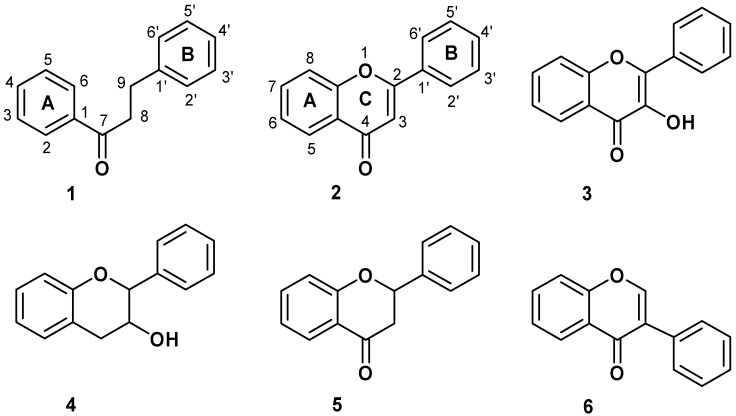
Structure and numeration of flavonoids derivatives.

**Figure 2:**
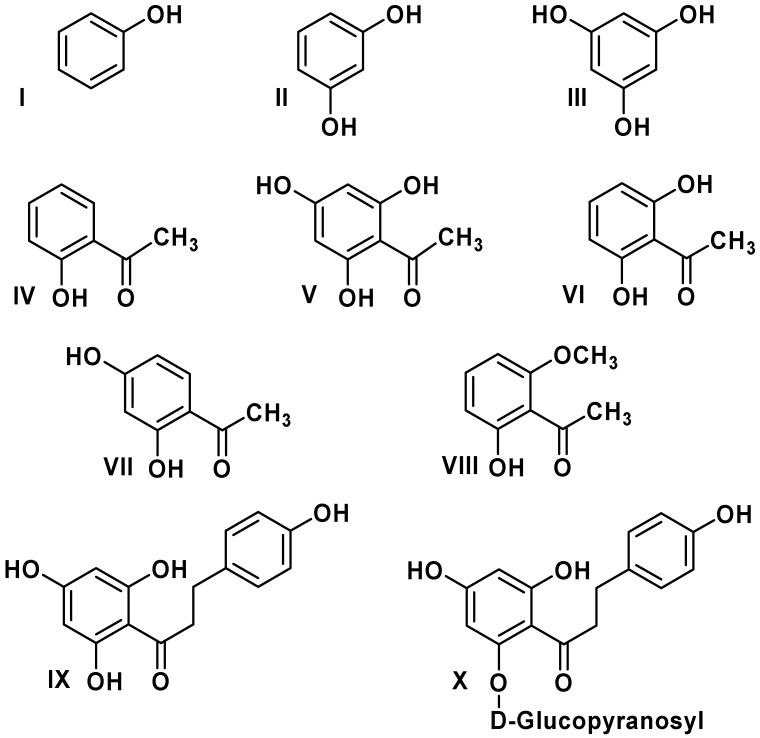
Structure of dihydrochalcones and related derivatives.

Antioxidants are of great interest because of their role in important biological and industrial processes. Flavonoids are the most abundant natural antioxidants, as well as abundantly present in green vegetables, fruits, olive, red wine, chocolate, and tea [1]. The chemical structure of flavonoids is based on a fifteen carbon skeleton with a chromane ring bearing a second aromatic ring. The flavonoid subgroups are classified according to the C-ring substitution pattern, in addition to the oxidation state of the heterocyclic ring and the position of B-ring. Examples are: chalcones**1**, flavones **2**, flavonols **3**, flavanones **4**, anthocyanins **5**, and isoflavonoids **6** (Figure 1). Chalcones (1,3-diaryl-2-propen-1-ones) are flavonoids lacking a heterocyclic C-ring, and they have also a broad spectrum of bioactivities such as anticancer, antifungal, antibacterial, antiviral, antioxidant and anti-inflammatory properties [2,3,4]. Recently, Rezk *et al*. [3] have reported a series of dihydrochalcones and structurally related acetophenones with antioxidant activity (Figure 2).

In the literature, two main mechanisms are proposed to explain the protective role for antioxidants [5,6,7,8]: One is the H-atom transfer, in which a free radical R^•^ removes a hydrogen atom from the antioxidant (ArOH) Equation (1):

R^•^ + ArOH → RH + ArO^•^ (1)

and the other one is a one-electron transfer mechanism, where the antioxidant can give an electron to the radical Equations (2) and (3):

R^•^ + ArOH → R^-^ + ArOH^•+^ (2)

R^•^ + ArO^-^ → R^-^ + ArO^•^ (3)

In addition to these mechanisms, the radicals arising from both reactions (ArO^•^ and ArOH^•+^) must be stable to prevent chain radical reactions.

Another not exhaustively studied mechanism is a consequence of their ability to chelate transition metal ions (M^n+^), which gives rise to stable complexes that prevent metals from participating in free radical generation [9]. As an example, during the Fenton reaction [10,11], hydroxyl radicals (HO^•^) are produced from hydrogen peroxide in the presence of a metal in a low oxidation state Equation (4):

H_2_O_2_ + M^n+^ → HO^-^ + HO^•^ + M^(n+1)+^ (4)

Many of these flavonoids contain a catecholic group in the B-ring and a resorcinolic group in the A-ring. When the radical is derived from catechol, it can be stabilized by an *ortho*-hydroxyl group and intramolecular hydrogen bonding [12]. However, the B-ring has been recognized as the active center of flavonoid free radical scavenging [13]. Nevertheless, quercetin seems to be a paradox since highly reactive species are neutralized during the same process, and reactive oxidation products are formed in the catechol group. The primary oxidation product of quercetin will be a semiquinone radical and second oxidation reaction will yield a quercetin–quinone. It was found recently that the formation of reactive quinone-type electrophiles from flavonoids is of importance for the understanding several beneficial and toxic effects on these systems [14].

On the other hand, few structure-activity relationship (SAR) investigations have been performed on the antioxidant activity of chalcone derivatives. Kozlowki *et al*. [15] have demonstrated the structural requirements for the 2,2-diphenyl-1-picrylhydrazyl (DPPH) free radical-scavenging properties of chalcones by H atom transfer, which include a catechol moiety at the C_3’_ and C_4’_ positions in the B-ring, a C_8_ = C_9_ double bond, and a free OH at the C_3_ position. However, interest in the SAR of dihydrochalcones and related derivatives with regard to their scavenging of oxygen and nitrogen radical species is minimal. The purpose of this work was twofold: one is to contribute to a better understanding of the mechanistic features of these processes in 2,4,6,4’-tetrahydroxy-dihydrochalcones and their derivatives against ROS and RNS, and the other is to determine the pharmacophore responsible for antioxidant activity of dihydrochalcones.

## 2. Results and Discussion

The stabilization energy (ΔE_iso_) is used as simple method to predict the ability of antioxidants to trap free radicals of phenolic derivatives. In Table 1 values of ΔE_iso_ are shown, and according to these values, it is possible to establish the following relative stability for the radicals at specified positions: an increase in the number of hydroxyl groups in the phenol A-ring increases the ΔE_iso_ due to the fact that more oxygen atoms of the phenolic hydroxyl groups can donate electrons to stabilize the semiquinone form. By the way, an addition of hydroxyl moiety in the *meta*-position decreases ΔE_iso_ and lower the scavenging effects.

**Table 1:** Stabilization energy of the phenoxy radicals II – X relative to I. The position of the most stable fenoxy radical in given in parenthesis.

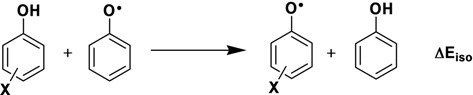			
Species	PhOH	PhO.	ΔE_iso_
	au	au	Kcal/mol
I	−307.478467	*−*306.835385	0.00
II	*−*382.698201	*−*382.056648	(*−*0.96)
III	*−*457.919066	*−*457.275369	0.39
IV	*−*460.143138	*−*459.472936	17.02
V (6)	*−*610.584324	*−*609.945225	(*−*2.50)
VI (6)	*−*535.362116	*−*534.723866	(*−*3.03)
VII (4)	*−*535.366132	*−*534.715737	4.59
VIII	*−*574.666026	*−*573.991339	19.83
IX (6)	*−*956.175410	*−*955.537571	(*−*3.29)
X (4’)	*−*1566.909704	*−*1566.267755	(*−*0.71)

Nevertheless, addition of an acetyl group in the *ortho*-position increases ΔE_iso_. The phenyl A-ring connecting two or three hydroxyls in *ortho*- and *para*-positions and a carbonyl group in phenolic derivatives may stabilize the radical formed during oxidation, extending the conjugation via resonance effects and contributing to the increased ΔE_iso_.

On the other hand, methoxylation in the *ortho*-position does not seem to be important for ΔE_iso_. As a consequence, molecules that showed several resonance structures were more stable and have higher ΔE_iso_ values. The absence of C_8_-C_9_ double bonds in the chalcone does not permit electronic conjugation between the C-ring and carbonyl group, or *s-cis* and *s-trans*-type isomerization compared with chalcone derivatives [15]. Nonetheless, molecules IX and X (Figure 2) are characterized by a great conformation flexibility and the fact that ΔE_iso_ values depend only on the A-ring or C-ring.

These results agree with biological tests since H_2_O_2_ can react with Fe, resulting in the formation of the highly reactive hydroxyl radical (HO^•^) via the Fenton reaction, while nitrogen centered reactive species can be produced by nitric oxide synthase (NOS). NOS catalyzes the synthesis of the radical species nitric oxide (NO) from the catalytic conversion of arginine to citrulline. The fastest known biological reaction for NO is the combination with superoxide to form a radical product peroxynitrite (ONOO^•^). Consequently, the compounds with higher ΔE_iso_ values will have more scavenging activities against HO^•^ and ONOO^•^ [5,6,7,8]. In fact, during oxidative stress, the conjugation and electronic delocalization depends from the number and positions of hydroxyls in resonance stabilization, especially in the case the dihydrochalcones. Therefore, low reactive species are formed between HO^•^ or ONOO^•^ and phenolic derivatives reaction after electron abstraction.

**Figure 3:**
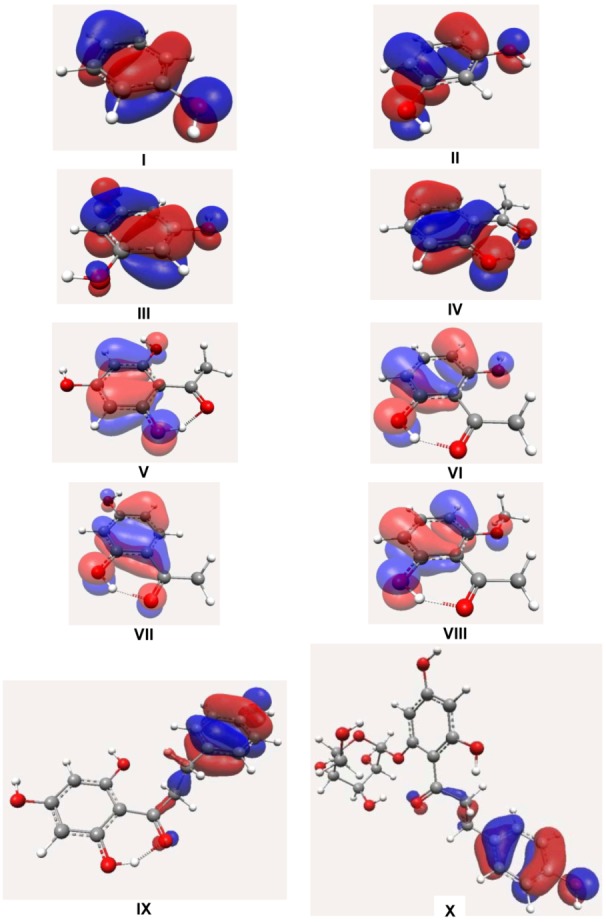
HOMO of dihydrochalcone-semiquinone and related derivatives.

The influence of hydroxylic and acylic groups on nucleophilicity of these compounds is much smaller on *para* and *meta*-positions of the A-ring. However, *ortho* orientations have good participation over π conjugation between both substituents. This behavior can be increased by participation of other hydroxyls at the 2- and 6-positions. These resonance effects can be observed in Figure 3. Nevertheless, a hydroxyl moiety at the *para*-position is important for conjugation over the B-ring. In fact, this is observed in compounds **IX** and **X**. The additional contribution of the B-ring is important for better scavenging activity, as shown in compound **IX**. The orientations of the hydroxylic groups affected by the extension of the π system change the energy in accordance with hydroxylic and acylic positions.

Values for bond dissociation enthalpy of hydroxyl (O-H BDE) and ionization potential (IP) are shown in Table 2.

The gas-phase O-H BDE for phenol has been determined by several experimental [16] and theoretical [17,18] methods, with the accepted value being 88.7 ± 0.5 kcal mol^−1^ [16]. The value found in our case, 81.87 kcal mol^−1^, is in fair agreement with the experimental results [16]. The IP value of 8.10 eV is also in agreement with the experimental result, 8.57 eV [19].

Therefore, experimental and theoretical methods have shown that phenol (**I**) has a small antioxidant activity. However, the introduction of 1 or 2 OH groups in *meta* position [forming resorcinol (**II**) or phloroglucinol (**III**), respectively], increases the antioxidant activity [3]. The introduction of OH groups decreases the IP when compared with phenol, resulting in better antioxidant activity. This result suggests that for these compounds the electron transfer mechanism is preferred for the scavenging process [17].

The introduction of a carbonyl group in the phenol (giving 2-hydroxyacetophenone, molecule **IV**) increases both IP and O-H BDE, resulting in a decrease in antioxidant activity when compared to molecules **I**, **II** and **III**. The introduction of two OH groups in molecule **IV** (giving molecule **V**) decreases both IP and O-H BDE only when the hydrogen at position C_6_ is considered for stabilization of the phenoxyl radical. Molecule **VI** also shows a decrease on both IP and O-H BDE when the hydrogen at position C_6_ is considered for radical stabilization whereas molecules **VII** and **VIII** show poor antioxidant activity. On the other hand, phloretin (molecule **IX**) has the lowest values for both IP and O-H BDE, so it should be the most active compound.

**Table 2:** IP and O-H BDE for dihydrochalcones and structurally related.

Compouds	BDE (O – H)	IP*^in gas^*	IP*^CPCM^*	PON	LPO
	(kcal mol^-1^)	(kcal mol^-1^)	(kcal mol^-1^)	IC_50_	IC_50_
**I**	81.87	186.66	137.28	552	>1,000
**II**	80.88	180.29	131.51	58	>1,000
**III**	82.02	178.14	133.20	39	624
**IV**	97.63	188.71	139.68	>1,000	>1,000
**V**-OH(2)	101.80	-	-	-	-
**V**-OH(4)	85.59	-	-	-	-
**V**-OH(6)	78.41	181.95	134.24	5.5	106
**VI**-OH(2)	99.76	-	-	-	-
**VI**-OH(6)	78.02	182.63	133.26	7.8	95
**VII**-OH(2)	99.58	-	-	-	-
**VII**-OH(4)	85.05	187.04	139.39	>1,000	>1,000
**VIII**	99.91	179.65	132.46	>1,000	>1,000
**IX**-OH(2)	98.38	-	-	-	-
**IX**-OH(4)	98.63	-	-	-	-
**IX**-OH(6)	76.32	167.48	132.86	3.1	24
**IX**-OH(4’)	78.63	-	-	-	-
**X** -OH(4)	82.50	-	-	-	-
**X** -OH(6)	77.40	-	-	-	-
**X**-OH(4’)	77.14	169.12	133.29	55	435

**Figure 4:**
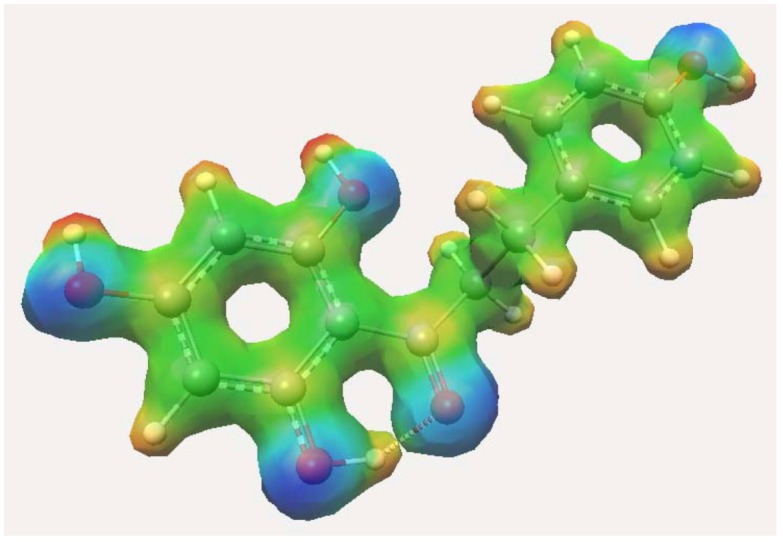
MEPs of dihydrochalcone derivative **IX**.

Kozlowski and co-workers have shown the importance of the double bond in the redox capacity. Its absence decreases the antioxidant activity [15]. Nevertheless, our results for molecule **IX** using B3LYP are in opposition compared with theoretical BDEs using B3P86. Theoretical BDEOH calculated using B3P86 for these authors shown that its values increase in the order of C4’ (84.1 kcal/mol), C2 and C6 (85.8 kcal/mol), and C4 (92.3 kcal/mol) positions. On the contrary, our results have been shown a BDEOH increase in the order of C6 (76.32 kcal/mol), C4’ (78.63 kcal/mol), C2 (98.38 kcal/mol), and C4 (98.63 kcal/mol) positions. These results have a direct influence of hydrogen bond between hydroxyl and carbonyl moiety, as shown in Figure 4, while the C4’ position is determined by the nucleophilicity of these molecules, such as HOMO of compounds **IX** and **X** (see Figure 3). In addition, this molecule also presents two possibilities to formation of radical at the C_6_ and C_4’_ positions. In phloridzin (**X**), one hydroxyl group was substituted by a sugar moiety. Phloridzin has higher values for both IP and O-H BDE and, as a consequence, it is less active. Similar values for O-H BDE have been found for flavonoids [18,19].

At least two mechanisms are involved in the radical scavenging processes for these chalcones: a direct H-atom transfer or an electron transfer process [17,20]. However, the two pathways co-exist in various chemical and biological systems. We can observe, with the exception of molecule **VII**, that in general the molecules with IP lower than 182 kcal mol^−1^ are more active, while molecules with IP higher than 186 kcal mol^−1^ are less active. For these 10 species, the theoretical IP in the gas phase is compared with IP calculated using polarized continuum medium (CPCM). The results in Table 2 show the IP by both calculation methods. IPs have a better correlation with antioxidant activity for all compounds using the two methods, while O-H BDE presents better correlation with antioxidant activity for compounds with a carbonyl group. In fact, we have observed few differences between the gas and water phase using the CPCM method. However, the IP values were decreased in the water phase using the CPCM method. For these compounds, the molecules with O-H BDE lower than 82 kcal mol^−1^ are more active, while molecules with O-H BDE higher than 99 kcal mol^−1^ are less active, suggesting that for these compounds the two pathways can co-exist. Recently, Zhang *et al*. [18] have found that 4-thiaflavans with values O-H BDE lower than 80 kcal mol are more active, in agreement with our results.

Nevertheless, the effectiveness of phenolic compounds depends not only on the stability of the phenoxyl radical formed in the reaction, but also on the substituents at different positions with respect to the phenolic group. For example, OH or OCH_3_ groups at the *ortho* position with regards to the phenolic group can stabilize a phenoxy radical by electron transfer to the electron-deficient radical site. Nonetheless, some flavonoid toxicity mechanisms are mediated by metabolism through cytochrome P-450 enzymes in the catechol group to form a reactive *ortho*-quinone metabolite. This metabolite reacts with glutathione (GSH) leading to its depletion or to its covalent binding to proteins. These events lead to increased production of reactive oxygen and nitrogen radical species, mitochondrial permeability transition and toxicity [21,22]. Therefore, the radical stability is important for the electron or hydrogen transfer of phenol derivatives [23,24].

**Figure 5:**
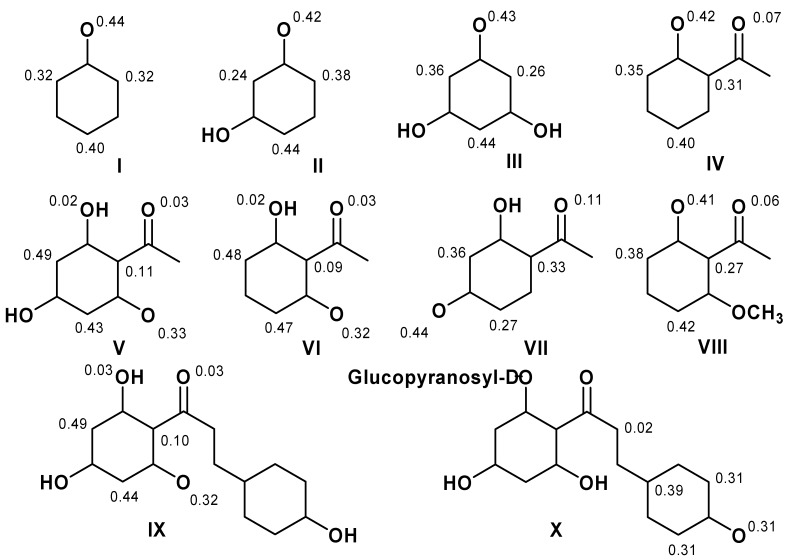
Spin densities for the more stable semiquinone derivatives.

In Figure 5, the calculated spin densities to hydrogen abstraction from the phenolic hydroxyl group showed that the phenolic oxygen contribution O_2_ is between 41–44%, and for phenolic oxygen O_6_, the phenolic oxygen O_4_, the C_3_ and C_6_ carbon atoms at *ortho* positions and the C_5_ carbon atom at *para* position are between 32–33%,_’_31%, 24–38%, and 40–49%, respectively. The lowest contribution of phenolic oxygen, being between 31–32%, showed the highest scavenging activity values due to the electronegativity of oxygen and to the fact that their compounds have more resonance spin structures.

The spin density is an important parameter to characterize the stability of free radicals, since the energy of a free radical can be efficiently decreased if the unpaired electrons are highly delocalized through the conjugated system after hydrogen abstration [25,26,27,28,29,30]. This agrees with Rezk [3], who states that a possible explanation for the potential antioxidant activity of 2,6-dihydroxyacetophenone might be found in the possible stabilization of the radical that is formed after hydrogen abstraction.

The prevalent contributions of HOMO, IP, BDEOH, ΔEiso, and spin densities are determinants for the biggest stable free radical and more resonance structures, and show that the π-type electron system of 2,6-dihydroxyacetophenone is the major antioxidant pharmacophore for dihydrochalcones. Therefore, we clarify the possible link between antioxidant activity of dihydrochalcones and 2,6-dihydroxyacetophenone, as its pharmacophore antioxidant.

## 3. Computational Methods

All the calculations were carried out using the Gaussian 03 programs [31], using the B3LYP/6-31G** level of theory [32,33]. The geometrical structures of the radicals studied were optimized independently from the neutral molecules prior to the calculations of spin densities and energies, and all of them are free from negative frequencies. The radicals were treated as open shell systems and the zero point vibration energies were scaled by a factor of 0.9805. The O-H bond dissociation enthalpies (O-H BDE), for homolytic O-H bond cleavage in the gas phase at 298.15 K, were calculated as the enthalpy of the radical (Hr) resulting from the hydrogen atom abstraction plus the enthalpy of hydrogen atom (H), -0.49765 Hartree, and minus the energy of the parent molecules (Hp); O-H BDE = Hr + H – Hp. The IP in the gas and water phase were calculated using the CPCM method, as the energy difference between a radical cation (Ec) and the respective parent molecule (Ep): IP= Ec – Ep, including the thermal correction for energy. The antioxidant activity of the 10 compounds was expressed as the concentration of the compound that gives 50% scavenging of the peroxynitrite (PON) radical and the concentration needed to inhibit 50% of the lipid peroxidation (IC_50_LPO) [3].

## 4. Conclusions

In this work, the antioxidant pharmacophore structure of the 2,4,6-trihydroxydihydrochalcones was investigated theoretically at the DFT/6-31G** level of theory. The hydroxyl groups have great importance in the resonance stabilization. The relative stability for the radical forms depends on specific positions, like hydroxyl groups in the *ortho* and *para*-positions or an acetyl moiety in the *ortho*-position, and contributes to the resonance effect. The introduction of hydroxyl and carbonyl groups decreases the IP when compared to phenol, resulting in better antioxidant activity. The electron (IP) or hydrogen donations (O-H BDE) are related to peroxynitrite scavenging activity and lipid peroxidation. The phenolic oxygen with lowest spin density contribution has the highest scavenging activity values. Results show that pharmacophore group depends on the substitution of hydroxyl groups in dihydrochalcones and acetophenone skeletons. In other words, it depends on the presence of a C_2_, C_6_, and C_4’_ hydroxyl moiety.
